# The association of maternal age with adverse neonatal outcomes in Lusaka, Zambia: a prospective cohort study

**DOI:** 10.1186/s12884-020-03361-5

**Published:** 2020-11-11

**Authors:** Tannia Tembo, Aybüke Koyuncu, Haoran Zhuo, Martha Mwendafilumba, Albert Manasyan

**Affiliations:** 1 Department of Reproductive, Maternal, Newborn and Child Health, Centre for Infectious Disease Research in Zambia, P.O Box 34681, Lusaka, Zambia; 2 Analysis Unit , Centre for Infectious Disease Research in Zambia , P.O Box 34681, Lusaka, Zambia; 3 Surgical Outcomes and Epidemiology-Surgical Department , Yale University , Connecticut CT 06520 New Haven, USA; 4 Division of Neonatology School of Medicine , University of Alabama at Birmingham , Birmingham, USA

**Keywords:** Adolescents, adolescent pregnancies, neonatal, neonatal mortality, LMIC

## Abstract

**Background:**

Pregnancy among adolescents, whether intended or not, is a public health concern as it is generally considered high risk for both mothers and their newborns. In Zambia, where many women engage in early sexual behaviour or marry at a young age, 28.5% of girls aged 15–19 years were pregnant with their first child in the year 2013–2014. This study sought to explore associations between maternal age and neonatal outcomes among pregnant women in Lusaka, Zambia.

**Methods:**

This was a secondary analysis of data nested within a larger population-based prospective cohort study which was implemented in three government health facilities-two first level hospitals and one clinic in Lusaka, Zambia. Women presenting to the study sites for antenatal care were enrolled into the study and followed up for collection of maternal and neonatal outcomes at 7, 28 and 42 days postpartum. The study’s primary outcomes were the incidence of maternal and newborn complications and factors associated with adverse neonatal outcomes. Statistical significance was evaluated at a significance level of *P* < 0.05.

**Results:**

The study included 11,501 women, 15.6% of whom were adolescents aged 10–19 years. Generally, adolescence did not have statistically significant associations with poor maternal health outcomes. However, the risk of experiencing obstructed labour, premature rupture of membranes and postpartum hemorrhage was higher among adolescents than women aged 20–24 years while the risk of severe infection was lower and non-significant. Adolescents also had 1.36 times the odds of having a low birthweight baby (95% CI 1.12, 1.66) and were at risk of preterm birth (aOR = 1.40, 95% CI 1.06, 1.84). Their newborns were in need of bag and mask resuscitation at birth (aOR = 0.62, 95% CI 0.41, 0.93). Advanced maternal age was significantly associated with increased odds of hypertension/ pre-eclampsia (95% CI 1.54, 5.89) and preterm labour (aOR = 2.78, 95% CI 1.24, 6.21).

**Conclusions:**

Adolescence is a risk factor for selected pregnancy outcomes in urban health facilities in Lusaka, Zambia. Health care workers should intensify the provision of targeted services to improve neonatal health outcomes.

**Trial registration:**

Clinical trial number and URL: NCT03923023 (Retrospectively registered). Clinical trial registration date: April 22, 2019.

## Background

Over the years, there has been a dramatic shift in childbearing age, globally [1]. Whereas the trend toward delayed pregnancy is reported particularly in developed countries due to various reasons, including women’s pursuit of higher education, career advancement, delayed marriage, effective birth control and advances in assisted reproductive technology [2], adolescent childbearing is increasing in Low-Middle Income Countries (LMICs) [3]. Several studies have evaluated the effect of maternal age on pregnancy outcomes due to the rising trend in early and advanced maternal age and associated risks of adverse obstetric outcomes [1, 4].

Advanced maternal age at birth, defined as 35 years and older [5], has been found to be associated with gestational diabetes, pre-eclampsia, risk of assisted birth, obstructed labour, placenta previa, caesarian section, placenta abruption, preterm birth, low birthweight, low Apgar scores, small for gestation age infants, infection, intrauterine fetal death and increased perinatal mortality [6–9]. Pregnancy among adolescents, whether intended or not, is a public health concern as it is generally considered high risk for both mothers and their newborns [10]. Early pregnancy and childbirth are associated with increased incidence of similar poor obstetric and neonatal outcomes as those shown in older women [11].

The World Health Organisation (WHO) reports that an estimated 18 million girls aged 13–19 years give birth annually, translating into approximately 1 in 4 girls bearing children by the age of 19 [12]. Approximately 95 percent of these births occur in LMICs [13]. In Zambia, where many women engage in early sexual behavior or marry at a young age, 28.5 percent of girls aged 15–19 years were pregnant with their first child between 2013 and 2014 [14]. The occurrence of some pregnancy related risks seem to be lower in the adolescent group. For instance, the risk of pregnancy-related maternal death is about a third higher among 15–19 year olds than among 20–24 year olds [15] while stillbirths and newborn deaths are 50 percent higher among adolescent mothers than among women aged 20–29 years [12, 16]. Young women are also more likely to give birth to low birthweight babies and experience obstructed labour, fistula and premature birth than older women [17].

Studies suggest that pregnancy complications are more often the result of socioeconomic factors rather than the biological age of the mother [18, 19]. Besides negatively affecting the emotional and physical wellbeing of young women, adolescent pregnancies compromise their human rights and opportunity to realise their social and economic potential [11, 20]. Early pregnancies have also been attributed to poverty, poor school performance, low future expectations, having an adolescent mother, low utilisation and accessibility to birth control [16, 21].

Although there has been a steady but uniform decrease in adolescent pregnancies from 137 to 96 per 1,000 adolescents in LMICs, 96 to 40 per 1,000 in middle income countries, and 46 to 13 per 1,000 in high income countries, the prevalence in sub-Saharan Africa (SSA) still remains high [22, 23]. Consequently, young women continue to experience problems related to pregnancy and childbirth. This study sought to evaluate the association of maternal age with poor neonatal outcomes in Lusaka, Zambia.

## Methods

This was a secondary analysis of data nested within a larger population-based prospective cohort - the Preterm Resources, Education, and Effective Management for Infants (PREEMI) study - which was implemented using a step-wedge model. Between May 2015 and September 2017, data were collected for births recorded in three public health facilities – Chawama and Chipata First Level Hospitals and George Health Centre – and surrounding catchment areas in Lusaka, Zambia. Study sites were selected on the basis of their high number of births one year prior to initiation of activities. During the implementation period, trained study staff – Quality Improvement Nurses (QI Nurses) and Community Liaison Officers (CLOs) – registered all pregnant women residing within the defined catchment areas and followed them until 42 days postpartum.

The PREEMI program activities were implemented as part of routine obstetric care and aimed to reduce neonatal mortality through the introduction of proven cost-effective clinical interventions such as Helping Babies Breathe (HBB) and Kangaroo Mother Care (KMC). Additionally, the study sought to enhance obstetric services and reduce gaps between health facility and home births by providing medical supplies, onsite clinical mentorship, and strengthening linkages between health facilities and community structures. Maternity service users were not engaged in the development of this study.

Health facilities were open 24 hours a day, 7 days a week, provided antenatal care (ANC), labour and birth and postnatal services with at least one nurse-midwife on duty throughout the day. As part of the national scale-up plan of Essential Newborn Care (ENC) services, nurse-midwives were trained to identify obstetric complications, accurately administer antibiotics for treatment of neonatal sepsis, antenatal corticosteroids for preterm labour, chlorhexidine gel (7.1 percent) for cord care and provide neonatal resuscitation with bag and mask. At community level, activities were implemented by Safe Motherhood Action Groups (SMAGs), community-based volunteer groups that are established to serve as a critical link between communities and health facilities thus supporting the Ministry of Health’s (MOH) efforts to reduce critical delays associated with individuals' decision-making about seeking health care, including life-saving maternal and newborn services.

Members of respective SMAGs were trained in KMC, early identification of danger signs at any time during pregnancy or labour and birth and immediate referral to higher levels of healthcare for further management. They were responsible for household follow ups during the postnatal period, at seven, 28 and 42 days, and were supervised by the CLOs. During follow-ups, data were collected on home births, outcomes (i.e. live births, miscarriages, stillbirths), and early (7-day) and late (28-day) neonatal mortality as well as baseline clinical characteristics of both mothers and their newborns.

Since the PREEMI program used a health system strengthening approach to reduce the risk of severe morbidity and mortality due to preterm birth complications in the selected health facilities and their wider catchment areas, we provided targeted services to all pregnant women attending ANC services at the three implementation sites. Given that two of the three implementation sites were first level referral hospitals, they provided services to women living within and outside of the health facilities’ catchment areas. However, while all women received the same quality of care, only women who lived within the catchment area and met the study’s inclusion criteria were eligible for enrolment into the parent study. Women residing outside the catchment areas but were seeking a one-off ANC service at one of the study sites were excluded from the study.

To be eligible for inclusion in this analysis, pregnant women should have registered for their first ANC visit or given birth at any of the study sites between May 2015 and September 2017. In 2015, the three implementation sites recorded a combined total of 14,350 births, including patients seeking services from outside the facility catchment areas [24]. Given the role of the two referral hospitals, we anticipated that half of the births will be among women living outside the facility catchment area. Assuming a projected population growth of 5.4% annually, this study expected health facilities to record 15,125 and 15,942 births in years one and two of implementation, respectively. Considering that these statistics would include women from outside the health facilities’ catchment areas and based on the study’s inclusion criteria, the study was expected to enroll 40 percent of the expected births during each year of implementation (i.e. 6,050 and 6,377, respectively).

Using study-specific data collection tools, study staff collected data from enrolled participants at health facilities during ANC, labour and delivery and postnatal visits or in the community during outreach activities. At community level, data on miscarriages, stillbirth, neonatal and maternal outcomes were collected by the CLOs through existing community structures (i.e. neighborhood health committees (NHCs) and SMAGs) and submitted to the QI Nurse at each study site. To facilitate data collection in the communities, the CLOs had weekly meetings with community volunteers, SMAGs, and NHCs.

The QI Nurses were responsible for collecting data at the health facility level. For quality control and quality assurance purposes, the QI Nurses and CLOs reviewed all the data before submitting them to the data team for entry. On a monthly basis, trained data associates performed consistency and completeness checks before and during data entry and at the time of producing reports. All study staff were trained in Good Clinical Practice (GCP), the study protocol, data collection and data collection tools. As per guidelines, GCP refresher trainings were held every three years, while refresher trainings pertaining the study protocol and data collection were conducted on an annual basis.

To determine the association of maternal age with adverse neonatal outcomes, this study used the 13 Core Outcome Sets (COS) relevant to women’s and newborn health and shortlisted in the CROWN database [25]. The primary outcomes of interest for this analysis were adverse maternal and neonatal outcomes. Specifically, we examined the incidence of the following maternal health outcomes: death, obstructed labour, pre-eclampsia, transverse or oblique lie, severe infection, preterm labour, PROM and severe antepartum and postpartum haemorrhage. Severe infection was determined through clinical screening and, when available, diagnostic tests (i.e. C-Reactive Protein (CRP), blood culture). Antepartum haemorrhage was defined as bleeding from or in the genital tract, occurring from 22 weeks (> 500 g) of pregnancy and prior to the birth of a baby. Postpartum Haemorrhage (PPH) was defined as blood loss of 500 ml or more within 24 hours after birth [20].

The primary neonatal outcomes of interest included the occurrence of low birthweight (< 2500 grams), preterm birth (infant born before 37 weeks’ gestation), the need for resuscitation at birth, and neonatal mortality during the first 7 and 28 days after birth. Additional outcomes of interest included miscarriage and stillbirth. We also examined a combined category of outcomes indicative of the occurrence of at least one poor maternal health outcome, and similarly a combined category of outcomes for the occurrence of at least one poor neonatal health outcome. All outcomes were self-reported by the mothers and ascertained by cross referencing data in the health facility registers.

The association between maternal age and adverse maternal and neonatal health outcomes was examined using logistic regression, yielding odds ratios (ORs) as our effect measure of interest. Consistent with WHO definitions, adolescent pregnancy was defined as pregnancy among mothers 10–19 years of age [26]. The distribution of sociodemographic variables, stratified by maternal age (10–19 years, 20–24 years, 25–34 years, and ≥ 35 years) were also assessed. Since evidence suggests that young girls and women of advanced maternal age are at increased risk of complicated pregnancy, the odds of adverse maternal or neonatal outcomes in these two groups were compared to those in a reference group of 20–24 year-olds who were likely to have a safe childbirth [27, 28].

In addition, the analysis examined unadjusted univariate associations between maternal age and adverse maternal and neonatal outcomes using chi-squared tests and fisher’s exact tests, where appropriate. Separate logistic regression models were constructed to examine unadjusted and adjusted associations between maternal age and each outcome of interest. Adjusted models included covariates specified *a priori* for inclusion as potential confounders based on direct acyclic graph (DAG) based analysis and existing literature on predictors of maternal and neonatal outcomes, namely: maternal education, attendance to antenatal care, and parity. All analyses were conducted in STATA 15 (College Station, Texas) and statistical significance was evaluated at an alpha significance level of *P* < 0.05.

## Results

A total of 11,535 adolescents and women were enrolled into the study. However, this analysis was restricted to 11,501 mothers after the exclusion of 34 mothers with missing values for maternal age. Of those enrolled, 1,788 (15.6%) were aged 10–19 years old, 3,980 (34.6%) aged 20–24 years old, 4,651 (40.4%) aged 25–34 years old and 1,082 (9.4%) aged ≥ 35 years old. Characteristics of the study population by maternal age are presented in Table 1.

**Table 1:** Characteristics of Study Population

Characteristic		Total (*N* = 11,501)	Maternal Age			
			**10–19 (*n* = 1,788)**	**20–24 (*n* = 3,980)**	**25–34 (*n* = 4,651)**	**35+ (*n* = 1,082)**
		**N ( column % )**	**N ( column % )**	**N ( column % )**	**N ( column % )**	**N ( column % )**
**Highest Education**						
No formal education		434 (3.8)	16 (0.9)	94 (2.4)	229 (4.9)	95 (8.8)
Some school/Primary school		4,380 (38.1)	635 (35.5)	1,195 (30.0)	1,939 (41.7)	611 (56.5)
Secondary school		6,351 (55.2)	1,129 (63.1)	2,590 (65.1)	2,281 (41.0)	351 (32.4)
College		327 (2.8)	5 (0.3)	100 (2.5)	197 (4.2)	25 (2.3)
** Parity (mean, SD)**		1.6 (1.5)	0.1 (0.4)	0.8 (0.8)	2.2 (1.2)	3.9 (1.4)
** ANC visits (mean, SD)**		3.1 (1.0)	3.1 (1.0)	3.1 (1.0)	3.1 (1.0)	3.0 (1.0)
**Mode of Birth**						
Vaginal		10,879 (94.6)	1,661 (92.9)	3,751 (94.3)	4,432 (95.3)	1,035 (95.7)
C-Section		200 (1.7)	27 (1.5)	67 (1.7)	81 (1.7)	25 (2.3)
**Location of Birth**						
Health facility		10,644 (92.6)	1,643 (91.9)	3,697 (92.9)	4,304(92.5)	1,000 (92.4)
Home		489 (4.3)	51 (2.9)	146 (3.7)	234 (5.0)	58 (5.4)
Other		17 (0.2)	3 (0.2)	4 (0.1)	7 (0.2)	3 (0.3)
**Birth Attendant**						
Physician (Obstetrician/ Non-specialist)		263 (2.4)	28 (1.7)	92 (2.4)	106 (2.3)	37 (3.5)
Nurse/Nurse-midwife		10,408 (93.1)	1,618 (95.3)	3,604 (93.7)	4,194 (92.3)	962 (90.6)
Traditional birth attendant		14 (0.1)	2 (0.1)	1 (0.03)	9 (0.2)	2 (0.2)
Family or neighbor		457 (4.1)	47 (2.8)	136 (3.5)	219 (4.8)	54 (5.1)
Self-birth		28 (0.3)	2 (0.1)	8 (0.2)	11 (0.2)	7 (0.7)

*SD* Standard Deviation

^a^Numbers may not sum to column totals due to missing data

In the overall population, as well as within each maternal age group, over 90% of the sample had a vaginal birth assisted by a nurse or nurse-midwife. Less than 5% of adolescent mothers, as well as mothers in the reference age group of 20–24, gave birth at home. Chawama and Chipata first-level hospitals were the most prevalent locations of birth enrolled in this study (46.3%, 50.8%, 53.2%, and 52.0% by increasing maternal age category, respectively). Mean parity at enrolment increased with maternal age. Unsurprisingly, adolescent mothers had lower parity (mean = 0.1), as they were enrolled in this study at their first pregnancy that resulted in a viable birth.

The incidence of adverse maternal and neonatal health outcomes stratified by maternal age is presented in Table 2. Notably, the incidence of each adverse maternal health outcomes was < 3% in the study population as well as within each stratum of maternal age. The frequency of maternal death 42 days post-partum was very low, with only nine observed deaths among older mothers and no deaths among adolescents. Low birthweight was the most frequently occurring adverse neonatal outcome, with the highest incidence observed among adolescent mothers (12.8%, *p* < 0.001).

**Table 2:** Adverse Maternal and Neonatal Outcomes by Maternal Age^a^

**Characteristic**	**Total (*N*=11,501)**	**Maternal Age**				
		**10-19**	**20-24**	**25-34**	**35+**	***p***-value^a^
		**(*n*=1,788)**	**(*n*=3,980)**	**(*n*=4,651)**	**(*n*=1,082)**	
	**N ( column % )**	**N ( column % )**	**N ( column % )**	**N ( column % )**	**N ( column % )**	
**Maternal Outcomes**						
Maternal death	9 (0.08)	0 (0.0)	3 (0.08)	5 (0.11)	1 (0.09)	0.6
Obstructed or prolonged labour	244 (2.3)	46 (2.8)	83 (2.2)	92 (2.1)	23 (2.2)	0.4
Severe haemorrhage (Antepartum/ Postpartum)	48 (0.4)	3 (0.2)	10 (0.3)	26 (0.6)	9 (0.9)	0.009**
Pre-eclampsia	172 (1.6)	27 (1.6)	43 (1.2)	76 (1.7)	26 (2.5)	0.01*
Breech/transverse or oblique lie	58 (0.5)	5 (0.3)	12 (0.3)	31 (0.7)	10 (1.0)	0.01*
Severe infection	20 (0.2)	2 (0.1)	6 (0.2)	7 (0.2)	5 (0.5)	0.2
Preterm labour	123 (1.1)	19 (1.2)	29 (0.8)	58 (1.3)	17 (1.6)	0.05*
Preterm premature rapture of membrane (PPROM)	41 (0.4)	7 (0.4)	7 (0.2)	22 (0.5)	5 (0.5)	0.1
**Neonatal Outcomes**						
Low birthweight (<2500 grams)	1,034 (9.7)	205 (12.8)	322 (8.8)	403 (9.3)	104 (10.2)	<0.001**
Preterm birth	546 (4.9)	102 (6.0)	154 (4.0)	228 (5.0)	62 (5.8)	0.004**
Resuscitation needed at birth	643 (5.9)	80 (4.8)	242 (6.4)	273 (6.1)	48 (4.6)	0.03*
Miscarriage	32(0.3)	3 (0.2)	11 (0.3)	16 (0.4)	2 (0.2)	0.7
Stillbirth	155 (1.4)	26 (1.5)	45 (1.2)	63 (1.4)	21 (2.0)	0.2
Death in first 7 days after birth	121 (1.1)	18 (1.1)	42 (1.1)	48 (1.1)	13 (1.3)	1
Death in first 28 days after birth	145 (1.3)	19 (1.1)	51 (1.4)	58 (1.3)	17 (1.6)	0.7

^a^*P*-value from chi-squared test, or fisher's exact test if number of outcomes <5 *p<0.05, **p<0.01

In this study, we found significant associations between adolescence and neonatal outcomes of low birthweight, preterm birth, and the need for resuscitation at birth in unadjusted and adjusted analysis (Table 3). Adolescent mothers had 1.36 times the odds of having a low birthweight baby compared to mothers aged 20–24 years (95% CI 1.12, 1.66). Adolescence was also a risk factor for preterm birth, with adolescent mothers having significantly higher odds of preterm birth compared to mothers in the reference category (aOR = 1.40, 95% CI 1.06, 1.84). Maternal ages of 25–34 and ≥ 35 years were also significantly associated with odds of having a low birthweight baby and preterm birth (Table 3).

**Table 3:** Association Between Maternal Age and Adverse Perinatal and Neonatal Outcomes

**Total (*N*=11,501)**		**Crude Odds Ratio**	**95% CI**	**Adjusted Odds Ratio**	**95% CI**
**Neonatal**	**Maternal Age**				
Low birthweight (<2500 grams)	10-19	1.52**	(1.27, 1.84)	1.36**	(1.12, 1.66)
	20-24	1.00 (ref)	-	1.00 (ref)	-
	25-34	1.06	(0.91, 1.24)	1.26*	(1.05, 1.51)
	35+	1.18	(0.94, 1.49)	1.61**	(1.18, 2.20)
Preterm birth	10-19	1.53**	(1.18, 1.98)	1.40*	(1.06, 1.84)
	20-24	1.00 (ref)	-	1.00 (ref)	-
	25-34	1.27*	(1.03, 1.56)	1.44**	(1.12, 1.85)
	35+	1.49*	(1.10, 2.02)	1.80**	(1.20, 2.71)
Resuscitation needed at birth	10-19	0.74*	(0.57, 0.95)	0.71*	(0.54, 0.92)
	20-24	1.00 (ref)	-	1.00 (ref)	-
	25-34	0.95	(0.80, 1.14)	0.92	(0.74, 1.14)
	35+	0.70*	(0.51, 0.96)	0.62*	(0.41, 0.93)
Stillbirth	10-19	1.32	(0.81, 2.14)	1.22	(0.74, 2.02)
	20-24	1.00 (ref)	-	1.00 (ref)	-
	25-34	1.19	(0.81, 1.75)	1.40	(0.90, 2.19)
	35+	1.71	(1.01, 2.88)	2.35*	(1.15, 4.78)
Death in first 7 days after birth	10-19	0.98	(0.56, 1.70)	1.04	(0.59, 1.87)
	20-24	1.00 (ref)	-	1.00 (ref)	-
	25-34	0.97	(0.64, 1.47)	0.84	(0.51, 1.39)
	35+	1.13	(0.60, 2.12)	0.80	(0.34, 1.87)
Death in first 28 days after birth	10-19	0.85	(0.50, 1.44)	0.90	(0.52, 1.57)
	20-24	1.00 (ref)	-	1.00 (ref)	-
	25-34	0.96	(0.66, 1.40)	0.84	(0.53, 1.32)
	35+	1.2	(0.70, 2.12)	0.88	(0.41, 1.89)
Any adverse neonatal outcomes^a^	10-19	1.08	(0.92, 1.26)	0.99	(0.84, 1.16)
	20-24	1.00 (ref)	-	1.00 (ref)	-
	25-34	1	(0.89, 1.13)	1.09	(0.94, 1.26)
	35+	0.97	(0.80, 1.17)	1.10	(0.85, 1.41)

^a^ Occurrence of any one of the following adverse neonatal outcomes: low birthweight, pre-term birth, resuscitation needed after birth, miscarriage, stillbirth, death in the first 7 days after birth, death in first 28 days after birth *p<0.05, **p<0.01

Maternal adolescence was protectively associated with the need for babies to be resuscitated at birth (aOR = 0.71, 95% CI 0.54, 0.92). Babies born to mothers of advanced maternal age similarly had a lower odds of needing resuscitation at birth (aOR = 0.62, 95% CI 0.41, 0.93). Advanced maternal age was also significantly associated with odds of having a stillbirth (aOR = 2.35, 95% CI 1.15, 4.78). Using combined outcome categories, adolescence was not associated with odds of experiencing at least one adverse maternal outcome or at least one adverse neonatal outcome (Table 3).

In unadjusted and adjusted analyses using logistic regression models, adolescence did not have a statistically significant association with any poor maternal health outcomes considered in this analysis (Table 4). Advanced maternal age, however, was significantly associated with an increased odds of hypertension/pre-eclampsia and preterm labour in our study population. In adjusted analyses, mothers ≥ 35 years of age had 3.01 times the odds of experiencing hypertension/pre-eclampsia compared to mothers 10–19 of age (95% CI 0.91, 5.73). Maternal age ≥ 35 years was also a significant risk factor for preterm labour (aOR = 2.78, 95% CI 1.24, 6.21).

**Table 4:** Association Between Maternal Age and Adverse Maternal Outcomes

**Total (*N*=11,501)**					
		**Crude Odds Ratio**	**95% CI**	**Adjusted Odds Ratio**	**95% CI**
**Maternal Outcome**	**Maternal Age**				
Maternal death	10-19	Non-estimable	-	Non-estimable	-
	20-24	1.00 (ref)	-	1.00 (ref)	-
	25-34	1.41	(0.34, 5.9)	2.85	(0.57, 14.23)
	35+	1.21	(0.13, 11.61)	5.19	(0.32, 82.81)
Obstructed or prolonged labour	10-19	1.27	(.88, 1.83)	1.31	(0.89, 1.92)
	20-24	1.00 (ref)	-	1.00 (ref)	-
	25-34	0.94	(0.70, 1.27)	1.07	(0.75, 1.52)
	35+	1.00	(0.63, 1.59)	1.42	(0.76, 2.65)
Severe antepartum haemorrhage	10-19	0.45	(0.05, 3.89)	Non-estimable	-
	20-24	1.00 (ref)	-	1.00 (ref)	-
	25-34	2.22	(0.79, 6.22)	2.00	(0.61, 6.59)
	35+	2.89	(0.78, 10.79)	2.09	(0.31, 13.96)
Severe postpartum haemorrhage	10-19	1.36	(0.33, 5.71)	1.08	(0.2, 5.71)
	20-24	1.00 (ref)	-	1.00 (ref)	-
	25-34	2.56	(0.93, 7.04)	1.83	(0.5, 5.73)
	35+	3.62*	(1.05, 12.52)	1.64	(0.32, 8.45)
Hypertension/pre-eclampsia	10-19	1.43	(0.88, 2.33)	1.52	(0.91, 2.53)
	20-24	1.00 (ref)	-	1.00 (ref)	-
	25-34	1.51*	(1.04, 2.20)	1.70*	(1.11, 2.61)
	35+	2.21**	(1.35, 3.62)	3.01**	(1.54, 5.89)
Breech/transverse or oblique lie	10-19	0.95	(0.33, 2.69)	0.99	(0.31, 3.15)
	20-24	1.00 (ref)	-	1.00 (ref)	-
	25-34	2.21*	(1.13, 4.30)	1.35	(0.63, 2.91)
	35+	3.03*	(1.30, 7.02)	1.07	(0.34, 3.37)
Severe infection	10-19	0.76	(0.15, 3.75)	0.42	(0.05, 3.64)
	20-24	1.00 (ref)	-	1.00 (ref)	-
	25-34	1.0	(0.33, 2.96)	1.02	(0.30, 3.49)
	35+	3.0	(0.92, 9.90)	3.56	(0.62, 20.50)
Preterm labour	10-19	1.49	(0.84, 2.67)	1.23	(0.67, 2.27)
	20-24	1.00 (ref)	-	1.00 (ref)	-
	25-34	1.71*	(1.09, 2.68)	2.11**	(1.26, 3.52)
	35+	2.13*	(1.17, 3.90)	2.78*	(1.24, 6.21)
Preterm premature rapture of membrane (PPROM)	10-19	2.28	(0.80, 6.50)	2.11	(0.68, 6.51)
	20-24	1.00 (ref)	-	1.00 (ref)	-
	25-34	2.69*	(1.15, 6.30)	2.03	(0.77, 5.34)
	35+	2.58	(0.82, 8.16)	1.41	(0.32, 6.27)

*p<0.05, **p<0.01

## Discussion

In this study, the prevalence of adolescent pregnancy was 15.6%. The cohort included pregnant girls between the ages of 10–19 years. Generally, pregnancy outcomes among adolescents were similar to those observed among older women. However, this study observed a higher incidence of low birth weight infants, preterm births and the need for resuscitation at birth among adolescents. We did not find associations between adolescence and the odds of experiencing obstetric complications such as pre-eclampsia and preterm labour. Improved neonatal health outcomes could be attributed to the availability of support groups and scheduled visits to the households of adolescent mothers with premature infants.

When compared to developed countries, the rate of adolescent pregnancy in LMICs still remains high with a serious impact on maternal and newborn health outcomes. While other studies have defined ages for adolescents and older mothers differently and evaluated perinatal health outcomes among adolescents aged 15–19 years [10, 7, 29], this study explored obstetric complications in 1,788 adolescents aged 10–19 years old and 9,713 mothers ≥ 20 years old. In this study, the observed 15.6% prevalence of adolescent pregnancy at three health facilities during a two-year period is similar to that shown in studies conducted elsewhere in sub-Saharan Africa and Latin America [30, 31] but higher than that recorded in India and Pakistan [32].

In the United States, negative outcomes of young mothers have been associated with environmental aspects rather than exclusively to maternal age. Therefore, accessibility to quality ANC, culture, poverty and poor education and socioeconomic status can also affect the advancement of pregnancy and consequently, its outcome [33, 34]. When compared to older mothers, a higher proportion of adolescent mothers in this study had attained some basic education (i.e. grades 1–9) by the time of their first ANC registration. This was similar to evidence from other literature [35] and could be attributed to an increase in school enrolments and attendance among children in many countries [36]. Nonetheless, an early start to childbearing may greatly reduce educational and employment opportunities for adolescent mothers [18]. In this study, we also observed an increase in mean parity with an increase in age.

Contrary to other findings that show that adolescent mothers had a lower rate of regular check-ups and were less likely to have regular ANC visits during pregnancy due to the lack of awareness of their importance [30, 35, 37], this study results show that there was no difference in the total number of ANC visits attended by mothers in the adolescent age group and those aged ≥ 20 years old. On average, mothers in both groups attended at least three ANC visits during the course of their pregnancies. This could be attributed to the level of education attained by adolescent mothers, their knowledge of the importance of ANC during pregnancy, community education provided by the SMAGs and acceptance of the adolescent pregnancy by the immediate families and community. In addition, adolescents in this study were more likely to give birth at a tertiary hospital with the help of a nurse-midwife as compared to older mothers. These results are similar to those from India where the percentage of adolescents with an early antenatal booking and birth in a health facility was higher than that of older mothers in previously reported studies [38, 39].

Adolescent pregnancies are said to be significantly associated with adverse maternal and neonatal outcomes [35–37, 40]. However, in our findings, consistent with results from studies conducted in Nigeria and six other LMICs [6, 32], adolescence generally did not have a statistically significant association with poor maternal outcomes. While the risk of obstructed/prolonged labour, PROM and PPH were higher among adolescents as compared to women aged 20–24 years, we found a lower and non-significant risk of severe infection. In Sweden, a nation-wide population-based study showed that adolescents and women aged 20–24 years were less prone to perinatal lacerations and PPH exceeding 1000 ml [41].

Since obstructed/prolonged labour is indicative of emergency cesarean [42, 43], we assume that the risk of cesarean section was higher among adolescent mothers as compared to older mothers. This is contrary to findings documented by Pan and Chauhan, 2011 [44]. Some studies suggest that cesarean section and assisted birth rates may be lower among young mothers due to acceptance of pregnancy and early booking [6].

Consistent with previous studies [27, 36], pregnancy induced hypertension or pre-eclampsia was significantly associated with advanced maternal age. In adjusted analyses, the risk was 3.01 times higher in mothers ≥ 35 years of age as compared to mothers aged 20–24 years. Other studies suggest that the risk of pre-eclampsia in adolescent pregnancy is increased by excessive gestational weight gain and obesity [45]. In accordance with a previous study [46], the risk of miscarriage was the same between adolescents and mothers ≥ 35 years old but not significantly different for mothers aged between 20 and 34 years.

Prematurity and low birth are intrinsically linked, therefore, infants who are born premature and have a low birthweight are at a higher risk of neonatal morbidity and mortality. In our study, a higher risk of preterm labour and low birthweight were observed among adolescents than older mothers. Other studies suggest a decrease in the magnitude of risk as age increases [6, 9, 10, 30, 46]. The risk of prematurity and low birthweight are associated with biological immaturity and feto-maternal competition for nutrients, respectively [27].

When compared to other papers on adolescent pregnancy published in Zambia, this study’s dataset is larger and therefore a more comprehensive representation of pregnancy and obstetric complications among adolescents. However, since the data for this cohort was collected in an urban setting, they may not be representative of the prevalence of adolescent pregnancies in the country and particularly rural settings where the prevalence is said to be high.

This study’s dataset includes births recorded at both health facility and community levels. More than 90% of the births captured for this cohort were conducted at the health facility level, which adds to the clinical rigor of this study’s analysis. However, even though data were collected prospectively with a 99% follow-up rate for 7-, 28-, and 42-day neonatal and maternal outcomes using standardized data collection tools, the baseline clinical characteristics which were collected by trained SMAG members may not have been accurately and completely captured. The analysis controlled for these characteristics so as to have a more accurate assessment of the identified risk factors.

In addition, while we collected a variety of variables to be able to assess risk factors for poor neonatal outcomes, some required services such as hemoglobin and urine tests were not provided consistently as part of standard of care at the implementation sites. Consequently, this analysis was not able to determine to what extent the missing variables could have an association with poor neonatal outcomes. Lastly, the data collected on gestation age at initial ANC visit and enrolment were not confirmed through ultrasound thus, this analysis was not able to control the outcomes based on Gestational Age (GA) at first ANC enrolment.

## Conclusions

The evaluation of obstetric complications in a cohort of pregnant mothers facilitated the identification of the most common risks among adolescents. There are no marked differences between maternal and neonatal outcomes of adolescents and older mothers. In fact, there were more significant outcomes in the women older than 35 years. While the Zambian government continues to adapt and support the implementation of evidence-based interventions, health care workers must adhere to national guidelines for providing quality healthcare for mothers and their newborns during the continuum of care to ensure improved health outcomes must provide evidence-based.

## Data Availability

The dataset generated and analysed during the current study are available from the corresponding author on reasonable request.

## Abbreviations

ANC: Antenatal Care
CIDRZ: Centre for Infectious Disease Research in Zambia
CLOs: Community Liaisons Officers
DAG: Direct Acyclic Graph
ENC: Essential Newborn Care
GA: Gestational Age
HBB: Helping Babies Breathe
KMC: Kangaroo Mother Care
LDHO: Lusaka District Health Office
LMICs: Low-Medium Income Countries
MOH: Ministry of Health
NHRA: National Health Research Authority
NHCs: Neighbourhood Health Committees
OR: Odds Ratio
PPH: Postpartum Haemorrhage
PREEMI: Preterm Resources, Education, and Effective Management for Infants
PROM: Premature Rupture of Membranes
QI: Quality Improvement
SMAGs: Safe Motherhood Action Groups
UAB: University of Alabama at Birmingham
UNZA BREC: University of Zambia Biomedical Research Ethics Committee
WHO: World Health Organisation
ZDHS: Zambia Demographic Health Survey
